# Neuronox versus BOTOX in the Treatment of Post-Stroke Upper Limb Spasticity: A Multicenter Randomized Controlled Trial

**DOI:** 10.1371/journal.pone.0128633

**Published:** 2015-06-01

**Authors:** Han Gil Seo, Nam-Jong Paik, Shi-Uk Lee, Byung-Mo Oh, Min Ho Chun, Bum Sun Kwon, Moon Suk Bang

**Affiliations:** 1 Department of Rehabilitation Medicine, Seoul National University College of Medicine, Seoul National University Hospital, Seoul, Republic of Korea; 2 Department of Rehabilitation Medicine, Seoul National University College of Medicine, Seoul National University Bundang Hospital, Seongnam, Republic of Korea; 3 Department of Rehabilitation Medicine, Seoul National University College of Medicine, Seoul National University Boramae Medical Center, Seoul, Republic of Korea; 4 Department of Rehabilitation Medicine, Asan Medical Center, University of Ulsan College of Medicine, Seoul, Republic of Korea; 5 Department of Rehabilitation Medicine, Dongguk University Ilsan Hospital, Goyang, Republic of Korea; University of Toronto, CANADA

## Abstract

**Background:**

Botulinum toxin type A is widely used for treating spasticity. Neuronox (Neu-BoNT/A), a newly manufactured botulinum toxin a, has not yet been investigated for its efficacy and safety in the treatment of post-stroke upper limb spasticity.

**Objective:**

We evaluated the efficacy and safety of Neuronox (Neu-BoNT/A) compared with BOTOX (onabotulinum toxin A) for treating post-stroke upper limb spasticity.

**Methods:**

In total, 196 stroke patients with moderate to severe upper limb spasticity were randomly assigned to either Neuronox or BOTOX intervention. The wrist flexors were mandatory and elbow, finger, and thumb flexors were optional muscles to be injected. Assessments were performed at baseline and 4, 8, and 12 weeks after the intervention. The primary outcome measure was the change from baseline of the Modified Ashworth Scale (MAS) at the wrist flexors at week 4. Secondary outcome measures included the change of MAS at each visit, response rate, Disability Assessment Scale (DAS), Carer Burden Scale, and Global Assessment of treatment benefit.

**Results:**

Primary outcome measures were -1.39±0.79 and -1.56±0.81 in the Neuronox and BOTOX groups, respectively. The difference was within the noninferiority margin of 0.45 (95% upper limit=0.40). There were no significant differences between the groups in the secondary outcome and safety measures, except the change of the MAS at the elbow flexors at week 12 (-0.88±0.75 in the Neuronox group, -0.65±0.74 in the BOTOX group; *P*=0.0429). Both groups showed significant improvements in the MAS, DAS, and Carer Burden Scale at weeks 4, 8, and 12.

**Conclusion:**

Neuronox showed equivalent efficacy and safety compared with BOTOX for treating post-stroke upper limb spasticity.

**Trial Registration:**

ClinicalTrials.gov NCT01313767

## Introduction

Upper limb spasticity affects 20% to 60% of stroke patients according to severity and duration of the disease [1–3]. Spasticity can cause pain, deformity, and contracture and may lead to functional loss and limited participation [4]. Currently, botulinum toxin type A (BoNT-A) is widely used for the treatment of upper limb spasticity in stroke patients. Numerous studies have reported its effect on reducing spasticity as well as functional improvement in these patients [5–9].

Onabotulinum toxin A (BOTOX; Allergan Inc, Irvine, CA) has been approved for the treatment of upper limb spasticity in the United States and most EU countries, and abobotulinum toxin A (Dysport; Ipsen Ltd, Slough, Berkshire, UK) has been approved for the said indication in most EU countries but not the United States. Because of their different properties, their comparability has been a subject of debate [10]. Neuronox (Medytox Inc, Ochang-eup, Cheongwon-gu, Cheongju-si, Chungcheongbuk-do, Republic of Korea), also known as Meditoxin in Korea, is a newly manufactured BoNT-A (Neu-BoNT/A) that was developed to provide features close to onabotulinum toxin A [11]. Neuronox was tested in a murine model, and its effect on muscle force generation was equivalent to BOTOX [12]. A previous multicenter randomized controlled trial showed that Neuronox and BOTOX have equivalent efficacy and safety for the treatment of spastic equinus in children with cerebral palsy [13]. However, Neuronox has not yet been investigated in post-stroke upper limb spasticity.

The present study aimed to compare the efficacy and safety of Neuronox with BOTOX in the treatment of post-stroke upper limb spasticity.

## Materials and Methods

### Ethic statement

This multicenter randomized controlled trial was approved by the Ministry of Food and Drug Safety and the institutional review boards of Seoul National University Hospital (H-0303-101-011), Seoul National University Bundang Hospital (B-1101-120-006), Seoul National University Boramae Medical Center (06-2010-193), Asan Medical Center (2010–0886), and Dongguk University Ilsan Hospital (2010-1-86), and it was registered at ClinicalTrials.gov (http://clinicaltrials.gov/; identifier: NCT01313767). The study was performed in accordance with the principles of Good Clinical Practice and the Declaration of Helsinki. Written informed consent was obtained from each patient or legal representative before study enrollment.

### Supporting information

The protocol for this trial and the supporting CONSORT checklist are available as supporting information (S1 Protocol and S1 CONSORT Checklist).

### Study Design

This study was a randomized, double-blind, multicenter, active-drug-controlled, phase III clinical trial to compare the efficacy and safety of Neuronox versus BOTOX in the treatment of post-stroke upper limb spasticity. It was conducted at 5 university hospitals (Seoul National University Hospital, Bundang Seoul National University Hospital, Seoul National University Boramae Hospital, Asan Medical Center, and Ilsan Dongguk University Hospital) in the Republic of Korea between March 2011 and January 2012.

### Participants

Stroke patients with moderate to severe upper limb spasticity were recruited for the study. The inclusion criteria were age ≥20 years, at least 6 weeks since the last stroke, at least 2 points in the focal spasticity of the wrist flexors, at least 1 point at one or more elbow flexors or finger flexors as measured on the Modified Ashworth Scale (MAS) [14], and 1 targeted functional disability item (hygiene, dressing, limb position, or pain) with a rating of ≥2 on the Disability Assessment Scale (DAS) [15]. The exclusion criteria were neuromuscular junction disorder, any botulinum toxin injection within 3 months, phenol or alcohol injection or surgery in the target limb within 6 months, fixed joint/muscle contracture or severe muscle atrophy in the target limb, concurrent treatment with intrathecal baclofen, known allergy or sensitivity to study medication or its components, pregnancy or planned pregnancy, and breastfeeding. Physical, occupational, and splinting therapy and muscle relaxants and benzodiazepine medication had to be stable from 1 month before screening and during the study.

### Randomization

Eligible participants were randomly assigned to either BOTOX or Neuronox intervention using a block randomization size of 4 or 6 and an allocation ratio of 1:1 at each hospital. An independent pharmacist diluted the medication with normal saline and loaded it into syringes according to the randomization code. Syringes loaded with BOTOX or Neuronox were not distinguishable, because their colors were identical. Therefore, the physicians who administered the injection and evaluated the outcomes and the participants were all blinded to which drug was assigned throughout the study period. The assigned codes for the participants were kept in sealed envelopes until the scheduled follow-ups were completed for statistical analysis. Although the code was available in case of serious potential side effects, no such issues occurred.

### Intervention

One vial of BoNT-A (100 U) was diluted with 2 mL normal saline. Selection of muscles and doses to be injected was determined by the physician based on study guideline and clinical assessment. The wrist flexors including the flexor carpi radialis (15–60 U, 1–2 sites) and the flexor carpi ulnaris (10–50 U, 1–2 sites) were mandatory muscles to be injected. Other muscles in the target limb were injected if the MAS at those muscles was ≥1 point. The flexor digitorum superficialis (15–50 U, 1–2 sites), the flexor digitorum profundus (15–50 U, 1–2 sites), the biceps brachii (100–200 U, up to 4 sites), the flexor pollicis longus (0–20 U, 1–2 sites), the adductor pollicis (0–10 U, 1–2 sites), and the flexor pollicis brevis/opponens (0–10 U, 1–2 sites) were optional muscles to be injected. The maximal total dose of BoNT-A was 360 U. Experienced physicians performed the intervention under electrical stimulation or electromyographic guidance.

### Assessment

Participants underwent history taking, physical examination, vital sign check, and blood and urine laboratory tests at the screening visit. BoNT-A was injected into eligible participants at the treatment visit within 2 weeks from screening. Follow-up evaluations were conducted 4, 8, and 12 weeks after the intervention.

### Efficacy Measures

Outcome measures including the MAS, the DAS, and the Carer Burden Scale were evaluated from baseline to week 12. The MAS was evaluated at the wrist, elbow, finger, and thumb flexors. The DAS is a scale for assessing functional impairment commonly seen in patients with post-stroke upper limb spasticity [15]. The rater determines the extent of functional impairment for hygiene, dressing, limb position, and pain from 0 (no disability) to 3 (severe disability) by patient interview. For this study, the physician selected 1 target domain based on patient and caregiver interviews. The Carer Burden Scale consists of 4 items, cleaning palms, cutting fingernails, dressing, and cleaning under armpits, for measuring the impact of upper limb spasticity on the physical burden of the caregiver [5]. Each item is rated by a 5-point Likert scale from 0 (no difficulty) to 4 (cannot do the task). The Global Assessment of treatment benefit ranging from 1 (very good) to 4 (poor) was evaluated by both the physician and the patient/caregiver at week 12.

The primary outcome measure was the change of the MAS from baseline at the wrist flexors at week 4. Secondary outcome measures included the change of the MAS at the wrist flexors at weeks 8 and 12, the change of the MAS at the other muscles, the response rate at all injected muscles, the change of the DAS and the Carer Burden Scale, and the Global Assessment. For evaluating the response rate, a positive response was defined as a decrease of ≥1 point of the MAS.

### Safety Measures

All adverse events were recorded for safety purposes. The number and rate of adverse events, treatment-emergent adverse events, adverse drug reactions, and serious adverse events were presented and compared between intervention groups. An adverse drug reaction was classified as related or not related by the physician based on the relation between the event and the drug. Vital signs were checked at each visit. Physical examinations and laboratory tests were performed at each visit. Any abnormality or change was presented and compared between the groups.

### Sample Size and Statistical Analysis

This study was designed to test the noninferiority of Neuronox compared with BOTOX. The noninferiority margin was defined as 0.45 based on previous studies reporting the change of the MAS at the wrist flexor for BOTOX as -1.1, -1.6, and -1.66 points [6,16,17]. The sample size was calculated to give 80% power (α = 0.05, 2-tailed test). Considering a 20% dropout rate, the total sample size was estimated to be 196 patients.

Patients with efficacy data were included in the analysis based on intention-to-treat. The datasets were classified as safety set, full analysis set (FAS), and per-protocol set (PPS). The safety set included all data from the participants randomized to the interventions. The FAS excluded participants from the safety set who had no efficacy assessment or an inclusion criteria violation. The PPS included participants who underwent all the study procedures without any serious protocol violations. The main outcomes were analyzed from the FAS, and additional analysis from the PPS was also performed for the primary outcome measure. For efficacy measures, missing data of the FAS were imputed on the last observation carried forward. However, cases with efficacy data missing at week 4 were excluded in the FAS analysis, because these data could not be replaced by the data before the intervention.

The primary outcome measure was analyzed by a 2-sample *t*-test after a normality test. Secondary outcome measures were compared at each visit between the groups using the 2-sample *t*-test to assess the change of the MAS from the baseline, χ^2^ and Fisher’s exact test to assess the response rate and the Global Assessment of the treatment benefit, and the Wilcoxon’s rank-sum test to assess changes in the DAS and the Carer Burden Scale from baseline. The Wilcoxon signed rank test and the McNemar test were used to evaluate changes in the MAS and the response rate after week 4, respectively. The weighted Cohen’s kappa and the Stuart-Maxwell test were used to evaluate the inter-rater agreement and the differences in the Global Assessment between the physician and the patient/caregiver. For safety measures, the number of adverse events was compared between groups by χ^2^ and Fisher’s exact test. The laboratory test results, physical examination, and vital signs were also analyzed based on the type and normality of the variables. Paired *t*-test and Wilcoxon signed-rank test were employed to assess the change of variables from baseline in each group. All the normality tests were performed using the Shapiro-Wilk test. *P* values <0.05 were considered statistically significant.

## Results

**Fig 1:**
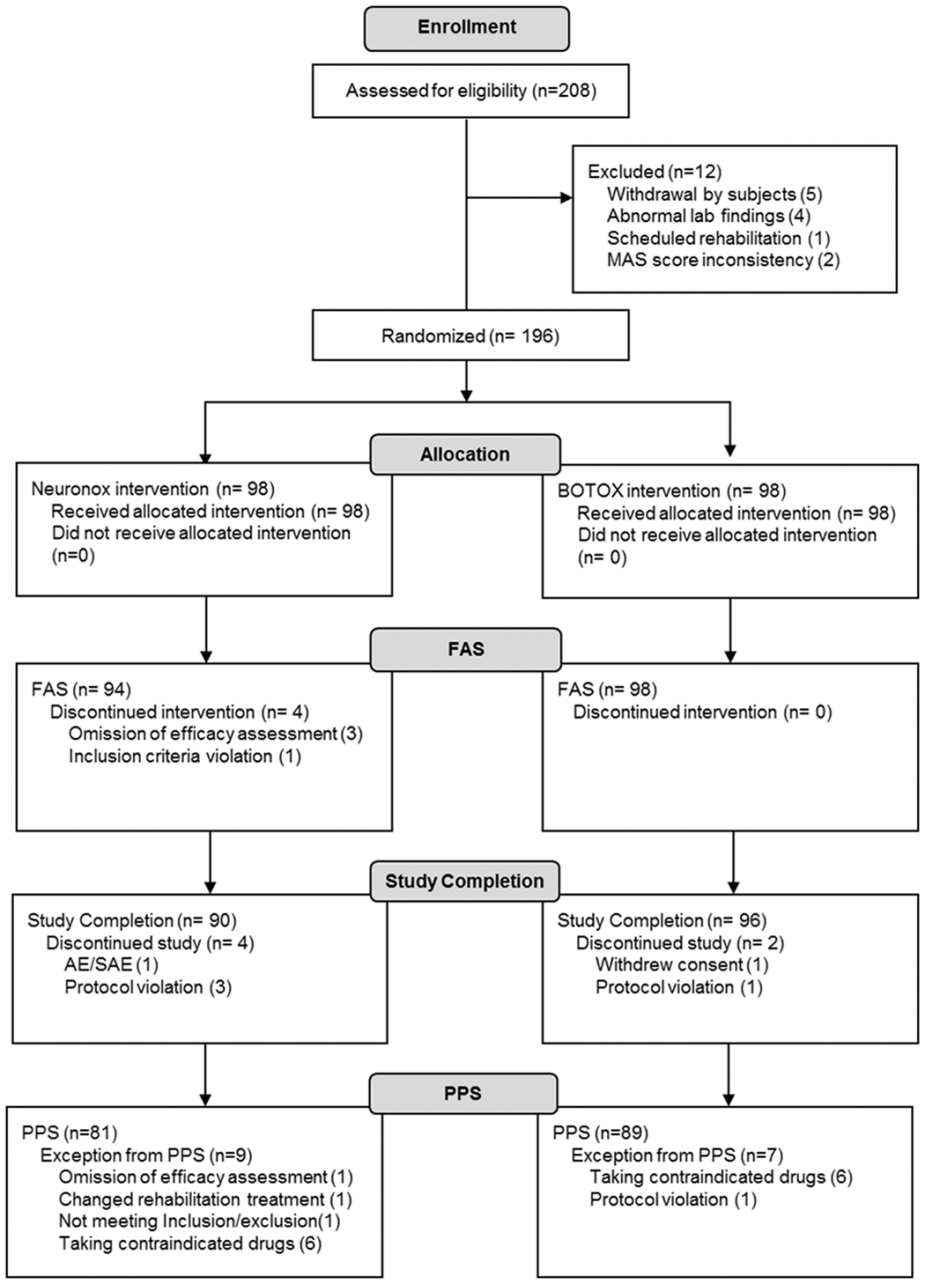
Study flowchart. MAS, Modified Ashworth Scale; FAS, full analysis set; PPS, per-protocol set.

Fig 1 shows the study flowchart. Of 208 eligible patients, 196 were randomly assigned to the Neuronox (n = 98) and BOTOX (n = 98) groups. FAS included 192 individuals after exclusion of 4 participants in the Neuronox group due to efficacy assessment omissions (n = 3) and inclusion criteria violation (n = 1). PPS included 81 in the Neuronox group and 89 in the BOTOX group. There was no significant difference in baseline characteristics between the groups (Table 1).

**Table 1:** Baseline Characteristics of Study Participants from the Full Analysis Set.

	Neuronox Group (n = 94)	BOTOX Group (n = 98)	*P*-value
Age (years)	57.54 ± 11.03	56.99 ± 13.01	0.7517*
Sex (n)			0.9070^†^
Men	65	67	
Women	29	31	
Weight (kg)	65.51 ± 10.27	62.94 ± 9.67	0.0771*
Time after stroke (months)	58.28 ± 61.19	58.45 ± 57.91	0.9441^§^
Previous BoNT-A injection (n)			0.1043^†^
Yes	20	31	
No	74	67	
Time from the last BoNT-A injection (months)	16.92 ± 20.76	18.37 ± 22.53	0.8926^§^
Current physical therapy (n)			0.8201^†^
Yes	60	61	
No	34	37	
MAS, wrist flexor (n)			0.5140^†^
2	61	56	
3	27	33	
4	6	9	
DAS score (n)			0.8780^†^
2	49	50	
3	45	48	

Values are presented as mean ± standard deviations and the number (n) of patients.

* Two-sample t-test

^†^ χ^2^ test

^§^ Wilcoxon’s rank-sum test

BoNT-A, botulinum toxin type A; MAS, Modified Ashworth Scale; DAS, Disability Assessment Scale

### Primary Outcome

In the FAS, the changes of the MAS from baseline at the wrist flexors at week 4 were -1.39±0.79 and -1.56±0.81 in the Neuronox and BOTOX groups, respectively (Table 2). The difference between the changes was 0.17, and the higher bound of the 95% confidence interval of the difference was 0.40, which was within the noninferiority margin of 0.45. In addition, there was no significant difference of the changes between the groups (2-sample t-test: *P* = 0.1347). Similar results were observed in the PPS (data not shown).

**Table 2:** Changes in the Wrist Flexor Spasticity Measured by the Modified Ashworth Scale at Week 4.

	Neuronox Group (n = 94)		BOTOX Group (n = 98)		Difference [95% CI]	*P*-value
	Mean ± SD	Median [IQR]	Mean ± SD	Median [IQR]		
Baseline	2.41 ± 0.61	2 [2, 3]	2.52 ± 0.66	2 [2, 3]		
Week 4	1.02 ± 0.81	1 [0, 1.5]	0.96 ± 0.64	1 [1, 1.5]		
Change	-1.39 ± 0.79	-1 [-2, -1]	-1.56 ± 0.81	-1 [-2, -1]	0.17 [-0.05, 0.40]	0.1347*

* Two-sample t-test.

SD, standard deviation; IQR, interquartile range; CI, confidence interval; FAS, full analysis set; PPS, per-protocol set.

### Secondary Outcomes

#### Modified Ashworth Scale

**Fig 2:**
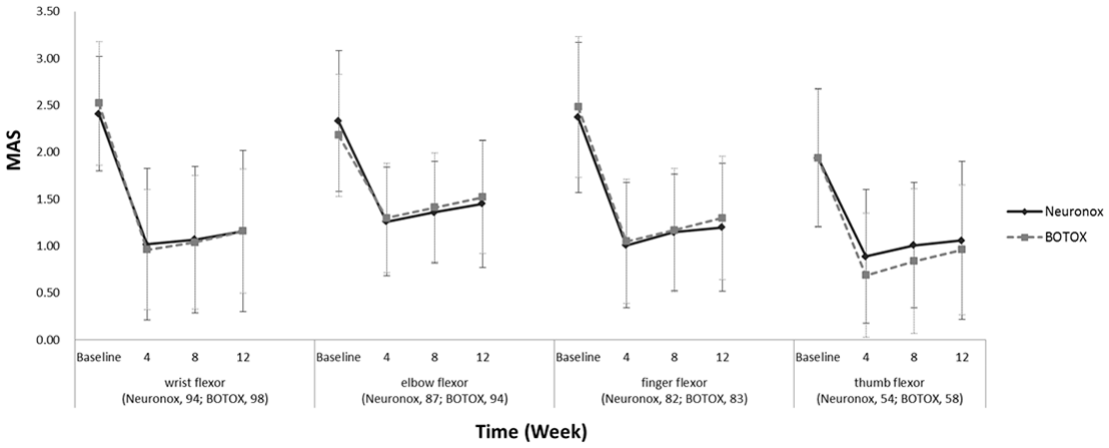
Changes of Modified Ashworth Scale (MAS) at each muscle group.

Fig 2 shows the changes of MAS at each injected muscle group. The MAS at weeks 4, 8, and 12 were significantly decreased from baseline at all injected muscles in both Neuronox and BOTOX groups (*P*<0.0001 by paired *t*-test). The treatment effect was decreased after week 4 in both groups. In the Neuronox group, the effect began to decrease at week 12 in the wrist and elbow flexors and at week 8 in the finger flexors (Wilcoxon signed rank test: *P* = 0.0444, 0.0128, and 0.0181, respectively). There was no significant difference from week 4 to weeks 8 and 12 in the thumb flexors. In the BOTOX group, the effect began to decrease at week 12 in the wrist flexors and at week 8 in the elbow, finger, and thumb flexors (Wilcoxon signed rank test: *P* = 0.0001, 0.0278, 0.0397, and 0.0101, respectively). The changes from baseline at weeks 4, 8, and 12 were not significantly different between the groups except at the elbow flexors. The changes of the MAS from baseline at the elbow flexors at week 12 were -0.88±0.75 and -0.65±0.74 in the Neuronox and BOTOX groups, respectively (*P* = 0.0429 by the 2-sample *t*-test).

#### Response Rate

**Fig 3:**
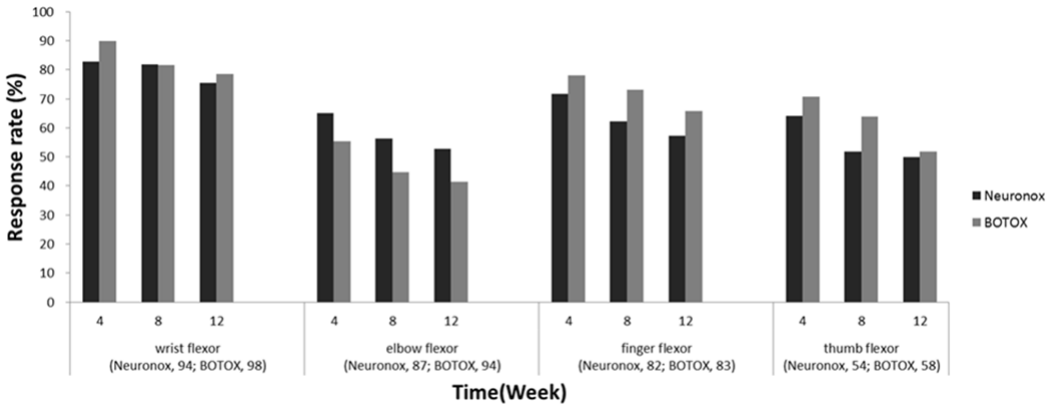
Response rates for each muscle group.

Fig 3 shows the response rates after BoNT injection, which at week 4 were more than 80% at the wrist flexors and ~60% at the elbow flexors. There were no significant differences in response rates between the groups. In the Neuronox group, the response rate began to decrease at week 8 in the elbow and finger flexors and at week 12 in the thumb flexors (McNemar test: *P* = 0.0114, 0.0348, and 0.0348, respectively). There was no significant change from week 4 to weeks 8 and 12 in the wrist flexors. In the BOTOX group, the response rate began to decrease at week 8 in the wrist and elbow flexors and at week 12 in the finger and thumb flexors (McNemar test: *P* = 0.0114, 0.0124, 0.0039, and 0.0045, respectively).

#### Disability Assessment Scale

There was no significant difference between the Neuronox and BOTOX groups in the changes of the DAS from baseline (Table 3). Both groups showed significant improvement from baseline at weeks 4, 8, and 12 in total and each domain (*P*<0.001 by Wilcoxon signed-rank test) except pain.

**Table 3:** Changes from Baseline according to the Disability Assessment Scale.

	Neuronox Group			BOTOX Group			*P*-value*
	n	Mean ± SD	Median [IQR]	n	Mean ± SD	Median [IQR]	
Total							
Week 4	93	-0.94 ± 0.73	-1 [-1, 0]	97	-0.96 ± 0.61	-1 [-1, -1]	0.9160
Week 8	94	-0.98 ± 0.67	-1 [-1, -1]	97	-0.98 ± 0.65	-1 [-1, -1]	0.9918
Week 12	94	-0.98 ± 0.73	-1 [-1, 0]	97	-1.01 ± 0.65	-1 [-1, -1]	0.8103
Hygiene							
Week 4	39	-0.72 ± 0.79	-1 [-1, 0]	34	-0.65 ± 0.65	-1 [-1, 0]	0.6040
Week 8	39	-0.77 ± 0.67	-1 [-1, 0]	34	-0.62 ± 0.65	-1 [-1, 0]	0.3223
Week 12	39	-0.74 ± 0.75	-1 [-1, 0]	34	-0.62 ± 0.60	-1 [-1, 0]	0.4469
Dressing							
Week 4	10	-0.70 ± 0.67	-1 [-1, 0]	14	-1.07 ± 0.47	-1 [-1, -1]	0.1220
Week 8	11	-0.73 ± 0.47	-1 [-1, 0]	14	-1.14 ± 0.66	-1 [-2, -1]	0.1016
Week 12	11	-0.82 ± 0.60	-1 [-1, 0]	14	-1.14 ± 0.66	-1 [-2, -1]	0.2235
Limb position							
Week 4	40	-1.23 ± 0.62	-1 [-2, -1]	47	-1.15 ± 0.55	-1 [-1, -1]	0.5030
Week 8	40	-1.23 ± 0.66	-1 [-2, -1]	47	-1.19 ± 0.54	-1 [-2, -1]	0.6934
Week 12	40	-1.20 ± 0.69	-1 [-2, -1]	47	-1.21 ± 0.55	-1 [-2, -1]	0.9574
Pain							
Week 4	4	-0.75 ± 0.50	-1 [-1, -0.5]	2	-1.00 ± 0.00	-1 [-1, -1]	0.7237
Week 8	4	-1.25 ± 0.50	-1 [-1.5, -1]	2	-1.00 ± 0.00	-1 [-1, -1]	0.7237
Week 12	4	-1.50 ± 0.58	-1.5 [-2, -1]	2	-2.00 ± 0.00	-2 [-2, -2]	0.4017

* Wilcoxon rank-sum test.

SD, standard deviation; IQR, interquartile range

#### Carer Burden Scale

There was no significant difference between the Neuronox and BOTOX groups in the changes of the Carer Burden Scale from baseline (Table 4). Both groups showed significant improvement from baseline at weeks 4, 8, and 12 in all items (*P*<0.05 by Wilcoxon signed-rank test).

**Table 4:** Changes from Baseline according to the Carer Burden Scale.

	Neuronox Group			BOTOX Group			*P*-value*
	n	Mean ± SD	Median [IQR]	n	Mean ± SD	Median [IQR]	
Cleaning the palm							
Week 4	90	-0.40 ± 1.29	0 [-1, 0]	98	-0.34 ± 1.10	0 [-1, 0]	0.8088
Week 8	92	-0.46 ± 1.24	0 [-1, 0]	98	-0.28 ± 1.09	0 [-1, 0]	0.3702
Week 12	92	-0.51 ± 1.34	0 [-1, 0]	98	-0.23 ± 1.17	0 [-1, 0]	0.1497
Cutting fingernails							
Week 4	90	-0.47 ± 1.26	0 [-1, 0]	98	-0.34 ± 1.17	0 [-1, 0]	0.9634
Week 8	92	-0.51 ± 1.29	0 [-1, 0]	98	-0.39 ± 1.25	0 [-1, 0]	0.7302
Week 12	92	-0.49 ± 1.35	0 [-1, 0]	98	-0.42 ± 1.19	0 [-1, 0]	0.7715
Putting the arm through a sleeve							
Week 4	90	-0.32 ± 1.12	0 [-1, 0]	98	-0.22 ± 0.98	0 [-1, 0]	0.9362
Week 8	92	-0.46 ± 1.11	0 [-1, 0]	98	-0.37 ± 1.17	0 [-1, 0]	0.7998
Week 12	92	-0.48 ± 1.13	0 [-1, 0]	98	-0.35 ± 1.15	0 [-1, 0]	0.5436
Cleaning under the armpit							
Week 4	90	-0.39 ± 1.12	0 [-1, 0]	98	-0.26 ± 1.20	0 [-1, 0]	0.7014
Week 8	92	-0.48 ± 1.12	0 [-1, 0]	98	-0.38 ± 1.26	0 [-1, 0]	0.8884
Week 12	92	-0.55 ± 1.19	0 [-1, 0]	98	-0.28 ± 1.43	0 [-1, 0]	0.2840

*Wilcoxon rank-sum test.

SD, standard deviation; IQR, interquartile range

#### Global Assessment of treatment benefit

The physician and patient/caregiver ratings were good or very good in 88.1% and 53.7% of patients in the Neuronox group and 77.3% and 55.7% of patients in the BOTOX group, respectively. There was no significant difference between the groups (Table 5). The inter-rater agreement between the physician and the patient/caregiver was poor in the Neuronox (weighted kappa: 0.1708) and BOTOX groups (weighted kappa: 0.1943). There were also significant differences in the assessment between the physician and the patient/caregiver in the Neuronox (*P* < 0.0001) and BOTOX groups (*P* = 0.0170), according to the Stuart-Maxwell test.

**Table 5:** Global Assessment of the Treatment Benefit by the Physician and the Patient/Caregiver at 12 Weeks after Injection.

	Neuronox Group (n = 93)	BOTOX Group (n = 97)	*P*-value*
Physician			0.2346
Very good	19 (20.4)	19 (19.6)	
Good	63 (67.7)	56 (57.7)	
Moderate	10 (10.8)	18 (18.6)	
Poor	1 (1.1)	4 (4.1)	
Patient/caregiver			0.9513
Very good	11 (11.8)	10 (10.3)	
Good	39 (41.9)	44 (45.4)	
Moderate	36 (38.7)	35 (36.1)	
Poor	7 (7.5)	8 (8.3)	

Values are expressed as the number of patients (%).

* Pearson’s χ^2^ test.

### Safety

Injected doses of BoNT-A were comparable between the Neuronox and BOTOX groups (Table 6). Adverse events were reported for 39 patients (93 events) in the Neuronox group and 41 patients (81 events) in the BOTOX group (*P* = 0.7713 by Pearson’s χ^2^ test). Adverse events occurring in at least 4% of patients were nasopharyngitis, extremity pain, and cough in the Neuronox group and upper respiratory tract infection and nasopharyngitis in the BOTOX group. Adverse drug reactions potentially related to the study treatment were reported in 4 patients (4 events) of the Neuronox group and in 8 patients (11 events) of the BOTOX group (Pearson’s χ^2^ test: *P* = 0.2334). These included an injection site hematoma, peripheral edema, pyrexia, convulsion, headache, hemiparesis, partial seizure, tendonitis, muscle weakness, increased alanine aminotransferase, and abnormal liver function test. Serious adverse events were reported for 5 patients in the Neuronox group (acute cholecystitis, toxic hepatitis, intraventricular hemorrhage, pneumonia, pulmonary tuberculosis, myocardial infarction, and renal failure) and 8 patients in the BOTOX group (convulsion, acute pyelonephritis, fall, femoral neck fracture, inguinal hernia, Behcet’s syndrome, and muscle weakness). One patient in the Neuronox group died because of myocardial infarction, pneumonia, pulmonary tuberculosis, and renal failure. Other patients recovered without sequelae. Among the serious adverse events, one (an abnormal liver function test) in the BOTOX group was considered potentially treatment related.

**Table 6:** Injected Doses of Botulinum Toxin Type A in a Safety Set.

	Neuronox Group			BOTOX Group			*P*-value*
	n	Mean ± SD	Median [IQR]	n	Mean ± SD	Median [IQR]	
Total	98	309.13 ± 65.49	350 [300, 360]	98	316.38 ± 54.70	350 [300, 360]	0.6603
Wrist flexor							
FCR	98	53.47 ± 6.44	50 [50, 60]	98	54.59 ± 5.59	50 [50, 60]	0.2727
FCU	98	47.30 ± 6.27	50 [50, 50]	98	48.57 ± 4.06	50 [50, 50]	0.1833
Elbow flexor							
Biceps	91	128.13 ± 29.13	120 [100, 150]	94	129.57 ± 29.58	120 [100, 150]	0.9842
Finger flexor							
FDS	83	44.10 ± 8.94	50 [40, 50]	81	44.75 ± 7.98	50 [40, 50]	0.7557
FDP	83	45.18 ± 9.12	50 [40, 50]	81	44.63 ± 9.28	50 [40, 50]	0.5869
Thumb flexor							
AP	21	13.81 ± 4.98	10 [10, 20]	23	14.13 ± 5.36	10 [10, 20]	0.8682
FPL	50	17.80 ± 4.18	20 [20, 20]	54	18.33 ± 4.23	20 [20, 20]	0.5443
FPB/FPO	17	10.00 ± 0.00	10 [10, 10]	16	10.00 ± 0.00	10 [10, 10]	1.0000

* Wilcoxon rank-sum test

SD, standard deviation; IQR, interquartile range; FCR, flexor carpi radialis; FCU, flexor carpi ulnaris; FDS, flexor digitorum superficialis; FDP, flexor digitorum profundus; AP, adductor pollicis; FPL, flexor pollicis longus; FPB, flexor pollicis brevis; FPO, flexor pollicis opponens

There was no significant difference between the groups in abnormal findings in vital signs and physical examinations. The only laboratory test changes from baseline at week 12 that differed significantly between the groups were in red blood cell count and hematocrit. The changes were considered clinically meaningless.

## Discussion

This randomized controlled trial showed equivalent efficacy of Neuronox and BOTOX on muscle tone, functional impairment, and caregiver burden in stroke patients with upper limb spasticity. Safety was also comparable between the 2 toxins. The >80% response rate in the wrist flexor suggested a sufficient spasticity reduction by the toxins.

The MAS at the wrist flexor changed -1.39±0.79 and -1.56±0.81 from baseline to week 4 in the Neuronox and BOTOX groups, respectively, comparable to previous results ranging from -1.1 to -1.66 [6,16–18]. The changes of MAS at the elbow and finger flexors were also similar to previous results: -0.9 to -1.2 at the elbow flexor [16,17] and -1.1 to -1.45 at the finger flexor [6,17,18]. A previous double-blind study using BOTOX to treat thumb flexor spasticity reported a mean reduction of MAS in the thumb flexor of -1.07 [6], also similar to our results. Response rates were >80% in both groups at the wrist flexor, comparable to or higher than previous results of 84.2% [18] and 62% [6].

The duration of the effect of BoNT-A in this study was also consistent with our knowledge of the toxin. BoNT-A induces reversible chemodenervation in the injected muscles through the abolition of acetylcholine exocytosis [19]. The functional paralysis induced by BoNT-A usually lasts for 3–4 months [20]. Although the therapeutic effect began to decrease after week 4, spasticity reduction by BoNT-A was maintained for 12 weeks after injection in both groups (Fig 2). The response rates at week 12 were approximately 80% at the wrist flexor, 60% at the finger flexor, and 50% at the elbow and thumb flexors. This finding was comparable to a seminal study in which the therapeutic effect of BoNT-A on wrist and finger spasticity was significant for 12 weeks after injection [6]. Although a significantly better effect of Neuronox was noted in the elbow flexors at week 12 compared to BOTOX, the overall deterioration in the therapeutic effect was similar in both groups.

The present study showed improved upper limb function measured by the DAS and Carer Burden Scale in both groups. Although BoNT-A has clearly reduced spasticity in stroke patients, there has been controversy over its effect on upper limb function. Several studies reported that BoNT-A reduced spasticity-associated disability in stroke patients [5–7]. However, a recent trial [9] suggested that BoNT-A may not improve active upper limb functions such as reaching and grasping in stroke patients with spasticity, although it may improve basic upper limb activities such as hand hygiene and dressing. Rousseaux et al [7] suggested that BoNT-A is efficient in improving hand use in patients with relatively preserved distal movements and in increasing comfort in patients with severe impairment. The results of this study were consistent with the previous findings on basic upper limb function. Further studies are warranted to elucidate the effect of BoNT-A on active function in patients with different upper limb impairments after stroke.

The BoNT-A injection treatment benefit was rated significantly higher by the physicians than by the patients and caregivers in the present study. It is hard to compare these results with those of previous studies, because the global assessment grades differed between studies [6,17,21]. In a study using a different BoNT-A formulation (NT 201) [22], treatment benefit was rated very good or good in ~60% of the patients after BoNT-A injection by the investigators, patients, and caregivers. In this study, the physicians may have been focused on changes of spasticity because the rate of more than good treatment benefit assessed by the physicians was similar to the response rates measured by the MAS. When assessing the treatment target and effect of BoNT-A, physicians should consider functional aspects of patients to lessen discrepancies between physicians and patients/caregivers.

A limitation of this study is that the BoNT-A was not compared with a placebo. The net effect of the study drug could not be determined by this study alone. However, there have been numerous placebo-controlled trials on the effect of BoNT-A, and treatment with BoNT-A injection has already been one of the standard treatments for patients with post-stroke upper limb spasticity. Therefore, placebo injection in these patients was considered ethically inappropriate, and the efficacy and safety of the study drug should be determined by comparison with a validated BoNT-A such as BOTOX.

In conclusion, the newly manufactured BoNT-A, Neuronox, showed equivalent efficacy and safety compared with BOTOX in the treatment of post-stroke upper limb spasticity. These results provide physicians with more options for BoNT-A injection for the treatment of spasticity in stroke patients.

## Supporting Information

S1 CONSORT Checklist CONSORT 2010 checklist of information to include when reporting a randomized trial. (DOC)

S1 Protocol A randomized, double blind, multi-center, active drug controlled, phase III clinical trial to compare the efficacy and safety of MEDITOXIN versus BOTOX in the treatment of post-stroke upper limb spasticity. (PDF)
