# Supplementary material for: Neuronox versus BOTOX in the Treatment of Post-Stroke Upper Limb Spasticity: A Multicenter Randomized Controlled Trial
Source: PLoS One. 2015 Jun 1;10(6):e0128633. doi: 10.1371/journal.pone.0128633 (PMC4452301; doi:10.1371/journal.pone.0128633)
Supplement: S1 Protocol — (PDF) [file pone.0128633.s002.pdf]

SYNOPSIS OF CLINICAL STUDY REPORT

Investigational Product: Meditoxin® Inj.

A randomized, double blind, multi-center, active drug controlled, phase III clinical trial to compare the efficacy and safety of MEDITOXIN® versus BOTOX® in treatment of post stroke upper limb spasticity

**Protocol No.**	**:**	**MT_PRT_ST01**
**Version No.**	**:**	**Ver 03.2_English ver 01**
**Report Date**	**:**	**17-Jun-2011**
**Sponsor**	**:**	**Medy-Tox, Inc.**

Confidentiality Statement

This protocol is the property of Medy-Tox and may not - in full or in part - be passed on, reproduced published or otherwise use without the express permission Medy-Tox. Do not disclose the information contained herein to others without written authorisation from Medy-Tox.

SYNOPSIS OF CLINICAL STUDY REPORT

**Study title**	A Randomized, Double Blind, Multi-center, Active Drug Controlled Clinical Trial to Compare the Efficacy and Safety of Meditoxin® versus Botox® in Treatment of Post Stroke Upper Limb Spasticity.
**Sponsor**	Medytox Inc.
**Institutions and Principal investigators**	**Institutions** / **Principal investigators**<br>Seoul National University Hospital, / Moon-seok Bang,<br>Asan Medical Center, , / Min-ho Chon<br>Seoul National University Bundang Hospital, / Nam-jong Baek,<br>Seoul Metropolitan Boramae Medical Center / Si-wook Lee<br>Dongguk University Ilsan Hospital / Beom-seon Kwon
**Objective**	To evaluate the efficacy and safety of the study drug (Meditoxin®), compared to controlled drug (Botox®) in treatment of post-stoke upper limb spasticity.<br><br>**Primary objectives**<br>Compare Meditoxin® vs. Botox® in change from baseline at week 4 for wrist flexor muscle tone as measured on the MAS (Modified Ashworth Scale) in patients with post-stoke upper limb spasticity<br><br>**Secondary objectives**<br>■ Compare the efficacy of Meditoxin® vs. Botox® in following outcomes:<br>• Change from baseline at week 4, 8, 12 for elbow flexor, finger flexor and thumb flexor muscle tone as measured on MAS<br>• Change from baseline at week 8 and 12 for wrist flexor muscle tone as measured on the MAS<br>• Percentage of treatment responders in wrist flexor, elbow flexor, finger flexor and thumb flexor at week 4, 8 and 12 after injection when a treatment response is defined as at least 1-point improvement from baseline on the MAS<br>•Change from baseline to week 4, 8 and 12 in a targeted domain of DAS(Disability Assessment Scale)<br>• Global assessment evaluated by investigator and subject/caregiver at week 12 after injection<br>• Change from baseline to week 4, 8 and 12 in the Carer Burden Scale evaluated by caregiver<br>■ Compare the safety of Meditoxin® vs. Botox®
**Study Design**	Double blind, randomized, active drug controlled, multicenter Phase III study
**Targeted Disease (Indication)**	Patients over 20 years old with post stroke upper limb spasticity
**Inclusion criteria**	**Inclusion Criteria**<br>1. Male or female patients ≥ 20 years<br>2. Diagnosed with stroke at least 6 weeks before the study enrollment<br>3. ≥ 2 points in the focal spasticity of wrist flexor and ≥ 1 points at least one of

	elbow flexor and finger flexor as measured on MAS<br>4. ≥ 2 points in one selected item among hygiene, dressing, limb position and pain for DAS (Disability Assessment Scale) assessment<br>5. Voluntary consent has been obtained with signed informed consent from by a subject or his/her legally acceptable representative.
**Exclusion criteria**	**Exclusion Criteria**<br>1. Systemic neuromuscular disorders (e.g. Lambert-Eaton syndrome, myasthenia gravis, or amyotrophic lateral sclerosis)<br>2. History of (within 6 months of IP treatment) or planned (during study period) treatment with phenol or alcohol injection or surgery in the target limb<br>3. History of (within 6 months of IP treatment) or planned (during study period) treatment with tendon lengthening in the target limb<br>4. Fixed joint/muscle contracture in the target limb Fixed joint or muscle contracture is defined as a condition with seriously limited mobility of joint due to significant resistance to passive movement.<br>5. Severe atrophy in the target limb (upper limb with spasticity)<br>6. Concurrent treatment with intrathecal baclofen<br>7. History of treatment with Botulinum Toxin within 3 months of IP treatment<br>8. Known allergy or sensitivity to study drug or its components (clorostridium botilinum toxin type A, human serum albumin or sodium chrolide).<br>9. For concurrent use of muscle relaxants and/or benzodiazepine medication, subjects who have changed the regimen within 1 month before screening; and subjects who are expected to change the regimen during the study period (12 weeks), even if these medications were stable from 1 month before screening<br>10. For a subject with current physical, occupational or splinting therapy in the target limb, these therapy has been changed within 1 month before screening or change is planned during the study period (12 weeks)<br>11. Subjects who are participating in other clinical study at the screening<br>12. Females who are pregnant or breast-feeding or have positive result in a serum or urine pregnancy test, or who do not agree to use of acceptable contraception from the screening up to 12 weeks after the end of treatment<br>* All females of childbearing potential should have negative result in a pregnancy test (urine or blood) conducted within 14 days of initial IP injection to participate in the study. Females who have had no menstruation for at least 12 consecutive or received surgical sterilization (bilateral tubal ligation, bilateral oophorectomy or hysterectomy) are not considered child-bearing potential. Medically acceptable contraceptions include specimide, contraceptive pill and barrier methods, intrauterine devices (IUDs) and complete abstinence.<br>13. Others determined inappropriate for the study in the investigator's opinion
**Number of subjects**	**Population Size (Planned Population Size and Actual Analysis Population Size)**
**Number of centers**	5 centers

Population Size (Number of subjects):

	Study Group (Meditoxin®)	Control Group (Botox®)	Total
Analysis Population Size	78	78	156
Analysis Population Size including drop-out (20%)	98	98	196

**Investigator Products**	**Experimental drug**<br>Meditoxin® (Clostridium Botulinum Toxin Type A, 100 unit) by Medytox Ltd.<br><br>**Controlled Drug**<br>Botox® (Clostridium Botulinum Toxin Type A, 100 unit) by Allergan Ltd.
**Fomula**	Injection with freezed dry white powder in colorless transparent vial
**Injection Site**	Intramuscular, IM
**Dosage · Administration**	Refer to the table below. Flexor carpi radialis and flexor carpi ulnaris related to wrist flexor have to be injected, but others are injected only the score of MAS is over 1. Selected sites can be injected up to total of 360U.

Injection Sites	Dosage	Injection Sites
**Flexed wrist**		
Flexor carpi radialis	15-60U	1-2 sites
Flexor carpi ulnaris	10-50U	1-2 sites
**Clenched fist**		
Flexor digitorum superficialis	15-50U	1-2 sites
Flexor digitorum profundus	15-50U	1-2 sites
**Flexed elbow**		
Biceps	100-200U	Up to 4 sites
**Thumb-in-palm**		
Flexor pollicis longus	0-20U	1-2 sites
Adductor pollicis	0-10U	1-2 sites
Flexor pollicis brevis/opponens	0-10U	1-2 sites

**Efficacy Variable**	(1) Primary efficacy endpoint:<br>Compare Meditoxin® vs. Botox® in change from baseline at week 4 for wrist flexor muscle tone as measured on the MAS (Modified Ashworth Scale) in patients with post-stoke upper limb spasticity<br><br>(2) Secondary efficacy endpoint :<br>■ Compare the efficacy of Meditoxin® vs. Botox® in following outcomes:<br>· Change from baseline at week 4 for elbow flexor, finger flexor and thumb flexor muscle tone as measured on MAS<br>· Change from baseline at week 8 and 12 for wrist flexor, elbow flexor, finger flexor and thumb flexor muscle tone as measured on MAS<br>· Percentage of treatment responders in wrist flexor, elbow flexor, finger flexor and thumb flexor at week 4, 8 and 12 after injection when a treatment response is defined as at least 1-point improvement from baseline on the MAS<br>· Change from baseline to week 4, 8 and 12 in a targeted domain of DAS(Disability Assessment Scale)<br>• Global assessment evaluated by investigator at week 12 after injection<br>■ Assessments by Subject or Caregiver<br>· Global assessment evaluated by subject/caregiver at week 12 after injection<br>· Change from baseline to week 4, 8 and 12 in the Carer Burden Scale evaluated

	by caregiver
**Safety Variable**	Adverse events, laboratory tests (hematological/blood chemistry tests, urine test), physical examination, vital signs
**Analysis Set**	Data obtained from subjects participating in the study are followed by ITT (Intention-to-treat) and categorized into Safety set, FAS (Full Analysis Set) and PP (Per-Protocol).<br><br>**[Efficacy evaluation set]**<br>Efficacy is analyzed in FAS and PP. In this study, FAS is the main analysis set for efficacy evaluation and additionally evaluation in PP is conducted.<br><br>**1) FAS (Full Analysis Set)**<br><br>Every enrolled subject who received randomized number is in FAS. However, the subject can be excluded from FAS if the subject as bleow;<br>• Subjects who are enrolled, but not treated with the investigational product<br>• Subjects who are treated with investigational product but never evaluated.<br><br>For any missing data due to subject's premature discontinuation or not-implemented procedure, LOCF (Last Observation Carried Forward Method) was employed in FAS analysis.<br><br>But, any missing data after IP treatment was not replaced with baseline value. Thus, when week 4 data was missing in primary and secondary efficacy endpoints, it cannot be replaced with baseline data and FAS analysis should be done with data missing, even though LOCF was used.<br><br>**2) PP (Per-Protocol) set**<br><br>FAS subjects who completed protocol without any protocol violation can be qualified for PP set. Subject exceptions in PP set analysis are followed in protocol section 12.5.<br><br>**[Efficacy evaluation set]**<br>Subjects with at least one IP treatment are in safety set. Safety evaluation is conducted for safety set.

Study timeline

Total of 5 visits:

Visit 1 (~ -14days): Screening

Visit 2 (week 0): Treatment

Visit 3 (week 4 ± 7 days), Visit 4 (week 8 ±7 days): Safety and Efficacy Evaluation

Visit 5 (week 12±7 days): Safety and Efficacy Evaluation, End of Study Visit

Visit 1 (~ -14 days)	Visit 2 (week 0)	Visit 3 (week 4 ± 7 days)	Visit 4 (week 8 ±7 days)	Visit 5 (week 12±7 days)
Screening Visit	Treatment	Follow-up Visit		End of Study Visit
		Safety and Efficacy Evaluation		

Statistical Analysis methods

Efficacy analysis methods

(1) Primary efficacy analysis

We provided 95% CI (both sided) for difference between study group and control group in change from baseline to week 4 in wrist flexor muscle tone as measures by MAS (Modified Ashworth Scale). If the upper limit of CI is no greater than 0.45 (non-inferiority margin), the study group is determined not inferior to the control group.

(2) Secondary efficacy analysis:

① Change from baseline at week 4 for muscle tone in the injected sites among elbow flexor, finger flexor and thumb flexor as measured on the MAS

Changes from baseline to week 4 in muscle tone of the injected sites among elbow flexor, finger flexor and thumb flexor are summarized with descriptive statistics (mean, standard deviations, median, minimum and maximum) by treatment group and the difference between groups is analyzed using two sample t- test.

② Change from baseline at week 8 and 12 for muscle tone in the injected sites among wrist flexor, elbow flexor, finger flexor and thumb flexor as measured on the MAS

Changes from baseline to week 4 in muscle tone of the injected sites among wrist flexor, elbow flexor, finger flexor and thumb flexor are summarized with descriptive statistics (mean, SD, median, minimum and maximum) by treatment group and difference between the two groups is compared using two sample t-test.

③ Percentage of treatment responders in wrist flexor, elbow flexor, finger flexor and thumb flexor at week 4, 8 and 12 after injection when a treatment response is defined as at least 1-point improvement from baseline on the MAS

We provided frequency and percentage of treatment responders who have improved at least 1-point on the MAS at week 4, 8 and 12 from baseline in wrist flexor, elbow flexor, finger flexor and thumb flexor and compared difference

	between the two groups using Pearson's chi-square test.<br>④ Change from baseline to week 4, 8 and 12 in the predefined target domain of the DAS(Disability Assessment Scale) evaluated by investigator<br>Changes from baseline to week 4, 8 and 12 in the predefined target domain of DAS are summarized with descriptive statistics (mean, SD, median, minimum and maximum) by treatment group and difference between the two groups is compared using Wilcox's rank sum test.<br>⑤ Global assessment evaluated by investigator at week 12 after injection<br>We provide frequency and percentage of each global assessment category measured at week 12 after injection by treatment group and compared difference between the two groups using Fisher's exact test.<br>⑥ Global assessment evaluated by subject/caregiver at week 12 after injection<br>We provide frequency and percentage of each global assessment category measured at week 12 after injection by treatment group and compared difference between the two groups using Pearson's chi-square test.<br>⑦ Change from baseline to week 4, 8 and 12 in the Carer Burden Scale evaluated by caregiver<br>Changes from baseline to week 4, 8 and 12 in Carer Burden Scale are summarized with descriptive statistics (mean, SD, median, minimum and maximum) by treatment group and difference between the two groups is compared using Wilcox's rank sum test.<br><br>**<u>Safety analysis methods</u>**<br>Adverse events (AEs), treatment-emergent adverse events (TEAEs), adverse drug reaction (ADRs) and serious adverse events (SAEs) occurred after obtaining consent are summarized with descriptive statistics (number of subjects, incidence rate and number of events) and any differences in incidences of AEs, TEAEs, ADRs and SAEs between groups are analyzed using Pearson's chi-square test or Fisher's exact test. AEs, TEAEs, ADRs and SAEs are coded using MedDRA by SOC (System Organ Class) and PT (Preferred Term) and the coded events are summarized with number of subjects, incidence rate and number of events by treatment group.<br>Continuous variables for clinical lab test, physical examination and vital sign are analyzed using paired-t-test or Wilcoxon's signed rank test depending on their fulfillment of normality assumption to determine intragroup changes from baseline to week 12, and categorical variables are analyzed using MacMemar's test. For changes from baseline to week 12 between groups, continuous variables are analyzed using two sample t-test or Wilcoxon's rank sum test and categorical variables are analyzed using Pearson's chi-square test or Fisher's exact test.
**Expected Study period**	1) Total study period (per subject): Approximately 12~15 months<br>2) Enrollment period: 10 months (Feb. 2011 ~ Nov. 2011)<br>3) Total study period (FPI~LPO): 13 months (Feb. 2011 ~ Feb. 2012)<br>* Total period can be modified by the speed of subject enrollment.
